# Deficits in narrative discourse elicited by visual stimuli are already present in patients with mild cognitive impairment

**DOI:** 10.3389/fnagi.2015.00096

**Published:** 2015-05-28

**Authors:** Cláudia Drummond, Gabriel Coutinho, Rochele Paz Fonseca, Naima Assunção, Alina Teldeschi, Ricardo de Oliveira-Souza, Jorge Moll, Fernanda Tovar-Moll, Paulo Mattos

**Affiliations:** ^1^D'Or Institute for Research and Education (IDOR)Rio de Janeiro, Brazil; ^2^Institute of Biomedical Sciences – Morphological Sciences Program, Federal University of Rio de JaneiroRio de Janeiro, Brazil; ^3^Department of Speech and Hearing Pathology, Federal University of Rio de JaneiroRio de Janeiro, Brazil; ^4^Laboratory of Clinical and Experimental Neuropsychology, Department of Psychology, Pontificial Catholic University of Rio Grande do SulPorto Alegre, Brazil; ^5^Department of Psychiatry and Forensic Medicine, Institute of Psychiatry, Federal University of Rio de JaneiroRio de Janeiro, Brazil

**Keywords:** narrative discourse, language, mild cognitive impairment, Alzheimer's disease, aging

## Abstract

Language batteries used to assess the skills of elderly individuals, such as naming and semantic verbal fluency, present some limitations in differentiating healthy controls from patients with amnestic mild cognitive impairment (a-MCI). Deficits in narrative discourse occur early in dementia caused by Alzheimer's disease (AD), and the narrative discourse abilities of a-MCI patients are poorly documented. The present study sought to propose and evaluate parameters for investigating narrative discourse in these populations. After a pilot study of 30 healthy subjects who served as a preliminary investigation of macro- and micro-linguistic aspects, 77 individuals (patients with AD and a-MCI and a control group) were evaluated. The experimental task required the participants to narrate a story based on a sequence of actions visually presented. The Control and AD groups differed in all parameters except narrative time and the total number of words recalled. The a-MCI group displayed mild discursive difficulties that were characterized as an intermediate stage between the Control and AD groups' performances. The a-MCI and Control groups differed from the AD group with respect to global coherence, discourse type and referential cohesion. The a-MCI and AD groups were similar to one another but differed from the Control group with respect to the type of words recalled, the repetition of words in the same sentence, the narrative structure and the inclusion of irrelevant propositions in the narrative. The narrative parameter that best distinguished the three groups was the speech effectiveness index. The proposed task was able to reveal differences between healthy controls and groups with cognitive decline. According to our findings, patients with a-MCI already present narrative deficits that are characterized by mild discursive difficulties that are less severe than those found in patients with AD.

## Introduction

Elderly individuals may seek treatment for a wide range of cognitive deficits. Because of the lack of specificity of these complaints, clinicians can have difficulty determining the real deficit. Difficulties associated with narrative discourse deficits, such as repetitions or information gaps during the narrative, are often the main reason for referral, but patients and relatives often attribute such deficits to memory problems. Because the traditional language tests used in clinical practice, such as naming and semantic or phonemic verbal fluency tests, do not address narrative discourse, such deficits may go unnoticed during evaluations.

Discourse is a complex linguistic activity that involves different levels of the linguistic system (phonological, morphosyntactic, semantic-lexical, and semantic-pragmatic) in conjunction with other cognitive aspects, including executive functions (Mar, 2004; Cannizzaro et al., 2012). Narrative discourse can be distinguished from other types of discourse—such as spontaneous and descriptive discourse—because it requires the speaker to verbally reproduce an episode experienced in the present (perception) or in the past (memory) while respecting the temporal and causal relationships among events that unfold in particular scenarios. This cognitive structure confers an ecological advantage to such evaluations (de Lira et al., 2011; Cannizzaro and Coelho, 2013).

Studies on narrative discourse have been conducted with individuals with right hemisphere injury (Marini et al., 2005; Fonseca et al., 2008; Ferré et al., 2011; Scherer et al., 2012a) and traumatic brain injury (TBI) (Davis and Coelho, 2004; Coelho et al., 2012). Difficulties with narrative discourse are already evident in the early stages of Alzheimer's disease (AD)-related dementia (Chapman et al., 2002; Duong et al., 2005; Mansur et al., 2005; Ska and Duong, 2005; Ash et al., 2007; Ferris and Farlow, 2013; Tsantali et al., 2013). However, little is known about the discursive characteristics of patients with amnestic mild cognitive impairment (a-MCI), which is a transitional stage between full cognitive ability and AD (Petersen, 2004).

Elderly individuals maintain the microstructural (phonological, lexical, and morphosyntactic) aspects of language and are able to understand discourse. However, they display a number of difficulties related to both the complexity of the task and the speed of the interlocutor's speech (Burke and Shafto, 2007; Peelle et al., 2010). An individual's discursive profile may be related to cognitive processes that usually decline with aging, such as processing speed, memory, attention (Wright et al., 2011), executive processes (Cannizzaro and Coelho, 2013), and visual perception or auditory processing (Bidelman et al., 2014). Several authors have suggested that changes in the discursive profile may also be associated with socio-affective aspects of adaptation to aging (Brandão and Parente, 2011), such as the need to reinforce one's identity (Lin et al., 2004). Repetitive and lengthy discourse with the addition of information or memories that reflect life experiences are among the most commonly described characteristics of elderly people's spontaneous discourse (Lin et al., 2004; Scherer et al., 2012b). At around the age of 70 years, specific difficulties with propositional content (Kemper et al., 2001) and spontaneous lexical access occur, such as slow word-finding, difficulty recalling names, and the “on the tip-of-the-tongue phenomenon” (Stamatakis et al., 2011). In contrast, lexical evocation via visual confrontation is preserved. The communicative difficulties of healthy aging are subtle (Madhavan et al., 2014) and stable and thus have less impact on daily functioning compared with the memory and executive changes that are present in normal aging (Scherer et al., 2012b; Caselli et al., 2014).

Even during the early stages of AD, individuals present linguistic difficulties (Teichmann and Ferrieux, 2013). In addition to the recognized difficulties with semantic lexical evocation (Henry and Crawford, 2004) and naming in response to visual stimuli (Lin et al., 2014), difficulties with narrative discourse occurs (Ash et al., 2007). Studies investigating narratives and recounting showed that AD patients have more deficits in macrolinguistic areas (semantic-pragmatic) than in microlinguistic ones (phonological, lexical and basic syntactic structure components) (de Lira et al., 2011). The discourse of AD patients is characterized by reduced information and less effective communication. The major difficulties might include exacerbated repetitions; a smaller number of propositions (Ska and Duong, 2005); difficulty reporting events in sequence (Mansur et al., 2005); information gaps that hinder overall meaning (Chapman et al., 2002; Mar, 2004); cohesion and overall coherence (Ash et al., 2007; Brandão and Parente, 2011); and difficulty making inferences. Such features could not be exclusively explained by difficulties with lexical access (Taler and Phillips, 2008).

a-MCI is clinically characterized by episodic memory deficits, but language impairment may also occur (Petersen, 2004). The literature suggests that confrontation naming and semantic verbal fluency tasks, regardless of the suggested category (Taler and Phillips, 2008), might be capable of differentiating patients with MCI from healthy older adults. However, there are some controversial findings. Some studies showed that the visual stimulus naming test is not suitable for detecting early AD (Testa et al., 2004) or distinguishing between normal controls and patients with MCI (Beinhoff et al., 2005). A study demonstrated that semantic verbal fluency tasks (naming animals and fruits) are useful for differentiating between normal elderly and AD groups but were less able to accurately differentiate the MCI groups from normal healthy elderly adults or people with AD (Radanovic et al., 2009; Lopez-Higes et al., 2014). There are few studies of narrative discourse in patients with MCI. Typically, these studies involve assessing narrative comprehension's demands on the memory domain by requiring patients to either recount or understand a heard story. The MCI group might exhibit difficulties in the global understanding of narratives; their performance is likely to be similar to that of the AD group and worse than that of the Control group (Chapman et al., 2002). The few studies that analyzed discourse production were based on single-scene description tasks, such as “The Cookie Theft Picture” task (Forbes-McKay and Venneri, 2005; Tsantali et al., 2013). To the best of our knowledge, no study has investigated the characteristics of the a-MCI group's narrative performance on narrative tasks using visual stimuli with sequences of actions.

Narrative production might be associated with different neuropsychological processes, such as episodic memory (Chapman et al., 2002; Taler and Phillips, 2008), executive function (Mar, 2004; Troiani et al., 2008; Cannizzaro and Coelho, 2013), and the semantic-pragmatic component of language (Fonseca et al., 2008; Troiani et al., 2008). Distinguishing the limits of these different functions is a challenge for clinicians and researchers. According to Lezak et al. (2012), it is very difficult to demarcate the boundaries of different but integrated cognitive processes.

The discourse tasks used to assess the narrative productions of elderly individuals are often based on an illustrated story without a text, a recounting of a heard story or a narrative describing a single picture (from a storybook or from a sequence of actions). The examined aspects might vary depending on the objectives and sample type of the study. Such studies typically consider macrolinguistic (i.e., related to planning, overall consistency, and coherence) or microlinguistic (i.e., related to words and sentences) aspects. Local and global coherence, referential cohesion, the narrative's structure and planning, the lexical index, the number and type of sentences, the generated propositions and the contextual suitability are among the most consistently analyzed criteria (Davis and Coelho, 2004; Ska and Duong, 2005; Ash et al., 2007; Wright et al., 2011; Coelho et al., 2012).

A review of the literature demonstrated the importance of using visual stimuli when evaluating individuals with AD (Davis and Coelho, 2004; Ska and Duong, 2005; Brandão and Parente, 2011). The use of tasks that involve describing a sequence of actions has advantages, such as requiring the participant to link facts, integrate scenes and establish the relationships among the events (unlike stimuli that present a single scene) and providing increased objectivity and reproducibility (compared with the analysis of autobiographical or spontaneous discourse). In addition, such tasks decrease the demand on episodic memory (Duong et al., 2003; de Lira et al., 2011).

As stated above, some studies have investigated discourse deficits among individuals with AD, but little is known about those deficits during the a-MCI stage. This issue is of clinical interest because a-MCI may constitute a predementia stage of AD (Petersen, 2011). As previously described, tests that elicit narrative discourse with visual stimuli could offer advantages such as avoiding memory overload, which could be a confounding factor for individuals with memory deficits such as a-MCI. To the best of our knowledge, no available tests use visual stimuli to assess this population.

The “car accident” task consists of seven scenes telling the story of an accident (Ska and Duong, 2005). The scenes are portrayed on cards that are presented to the individual, who is prompted to narrate the “story” without a time limit. This task was chosen because it involves a narrative situation that targets previously consolidated knowledge, that is, a common event that occurs in daily life. The number of scenes (seven) allows the narrative to be divided into three blocks of events (i.e., the initial event, the development and the outcome), thus permitting coherence and cohesion analyses that cannot be assessed with tests that present fewer sequences.

The present study has two objectives: (1) to introduce quantitative parameters for assessing the narrative discourse of the elderly based on a visually presented sequence of actions and (2) to investigate whether these parameters are able to distinguish among healthy controls, people with a-MCI and people with AD.

Based on the literature review, we hypothesized that narrative discourse deficits may be present in MCI because such deficits are already evident in the mild stages of AD.

## Materials and methods

Our study required a brief preliminary study (pilot study) because there are no data available in the literature regarding the psychometric properties of the task. The study had two phases. The first phase was exploratory and conducted with a non-clinical sample to define the parameters with which to quantify the narrative discourse based on visual stimuli (the car accident task (Ska and Duong, 2005), not standardized for the Brazilian population). The psychometric investigation of the task was beyond the scope of our study; however, we needed preliminary findings for a representative Brazilian sample that included individuals from both genders and different educational levels and ages. We needed to investigate how individuals narrate the story in our country; that is, we needed to determine which words and sentences were most often used in Portuguese to describe each of the seven scenes and which sentences were most often used to convey the main ideas of the story. The parameters of the pilot study were then used in the analyses of the second phase of our study. The second phase was the clinical application of these parameters to a-MCI and AD clinical groups. The Research Ethics Committee of the D'Or Institute approved this project (CEP 226/11). All of the participants provided written informed consent to participate in the study.

### Experiment 1: pilot study

#### Sample

Thirty individuals (males and females) ranging in age from 30 to 80 years were selected from a convenience sample. The sample was divided into two education levels (8 years or more and less than 8 years of education) to better represent Brazilian population. The individuals were recruited from the Federal University of Rio de Janeiro and the D'Or Institute of Research and Education via direct invitation. The sample comprised professors, undergraduate students, staff members and relatives of patients attending the university's outpatient unit. The participants were screened for global cognitive functioning using the Mini-Mental State Examination (MMSE). The participants with normal MMSE results based on Brazilian normative data (Brucki et al., 2003) were included. Three participants were excluded: two with more than 8 years of education and one with 2 years of education. All of the excluded volunteers had MMSE scores lower than expected for their age and education level.

#### Narrative task using visual stimuli

The participants were asked to narrate a story (the “car accident” task) based on seven scenes that were visually presented in the correct order. We explained that the story was a sequence of actions and checked to ensure that the individual clearly understood this. There was no time limit, and the following standardized instruction was given: *“Look carefully at this sequence of actions and tell me what you think happened.”*

All of the narratives were recorded on digital high-definition (HD) media and fully transcribed using semi-orthographic transcription. We transcribed exactly what the participant said, regardless of its grammatical correctness. This type of procedure is considered more suitable for the type of analysis we intended to perform because no interference or corrections occurred as the oral language was transposed into writing. All of the words, synonyms or equivalents were listed and compared, with the aim of identifying the keywords and central ideas (macropropositions) used to construct the narrative. All of the narratives were manually transcribed. After the transcription, we listed all of the open-class words that all of the participants used when telling the story. This procedure defined the most significant words related by group for each scene. Thus, we were able to define the keywords and central ideas used to construct the narrative. We obtained nine main ideas that were used to narrate the seven scenes (Supplementary Material).

#### Statistical analysis

Exploratory factor analysis (EFA) was used to extract the most relevant recalled words (i.e., “keywords”) and to identify the latent factors expressed by the scenes and keywords without the need to discriminate *a priori* the number of factors (keywords) to be identified. Next, confirmatory factor analysis (CFA) was used to define the groups of words that statistically related best to each core action (Supplementary Material). For the purposes of the pilot study and because of the limited sample size we decided to adopt a significance level (*α*) of 0.10, less stringent (Maas and Snijders, 2003; Gilani et al., 2013). This significance level was chosen to identify possible differences in load factor (scenes); using an (*α*) of 0.05 we could not be able to identify many “words” that could be important in different “scenes.” For these analyses, Mplus software, version 6.0, was used (Tucker and Lewis, 1973; Muthén and Muthén, 1998–2010). Some could argue that EFA was not the best method for this analysis because of the small sample size. Therefore, other analyses were performed. The Kaiser–Meyer–Olkin test (KMO) had a low value (0.55), but according to Schwab (2006) values above 0.5 allow factor analysis to proceed. The Bartlett test had a highly significant value (*p* < 0.001, indicating correlations among variables (“words”) and scenes.

#### Pilot study results

The mean age was 52.43 (± 17.7) years (range = 30–80 years), the average number of years of schooling was 10.7 (± 4.8; range = 4–16 years), and the MMSE score averaged 26 points (± 2.7; range = 21–30 points). There was a slight female predominance; the gender ratio was 18:12.

From the analysis of the 30 narrative transcripts, 101 open-class words (nouns, verbs, adjectives, and adverbs) with at least one occurrence were obtained. Through the EFA and the subsequent CFA, we identified 68 words, and their respective loadings related to nine aspects of the seven-scene story (Supplementary Material). For scenes two and six, two factors were related because they described two different, interdependent core actions. Supplementary Material presents the nine macropropositions. They were based on the nine story factors and 33 statistically significant words relative to each scene. The words (variables) showed significant correlations with their corresponding “scenes” (factors or latent variables) based on model fit (CFA).

Frequently occurring synonyms for or words and expressions related to the statistically significant words (those that appeared in the transcripts and were considered correct answers in this study) are also listed. The mean number of words used for the elaboration of each narrative was 97.4, and the mean time to complete the narratives was 51.3 s. The pilot study group averaged 7.1 (± 1.4) macropropositions (range 5–9). Regarding discourse type, there was a predominance of narrative discourse relative to descriptive discourse, with a ratio of 28:2.

Some results obtained from the pilot study (total number of words or micropropositions, time to execute the narrative or discourse type) were not used on the second phase of the study with clinical groups (Experiment 2) because the psychometric properties of the task needed to be further investigated. Furthermore, in experiment 2 we used a control group to compare all variables.

The initial experiment (pilot) was exploratory and aimed to define which words and which macropropositions were necessary for an adequate narrative telling the whole story.

### Experiment 2: clinical study

#### Sample

A total of 186 participants from a research project on diagnostic tools for AD-related dementia were evaluated. Of these, 77 met the inclusion criteria for the present study: age equal to or >60 years, education equal to or >8 years, and Brazilian Portuguese as the first language. The exclusion criteria were as follows: a clinical dementia rating (CDR) score higher than 1.0, frontotemporal dementia, primary progressive aphasia, dementia with Lewy bodies, advanced cerebrovascular disease, non-amnestic MCI or a major neuropsychiatric disorder, including major depression. We used the Geriatric Depression Scale (Brief Form) to screen patients for depression.

Most of the participants were referred by physicians, such as geriatricians, neurologists, and psychiatrists, from the City of Rio de Janeiro. The subjects were informed about the procedures and signed an informed consent form. All of the participants underwent a medical evaluation followed by a neuropsychological assessment, magnetic resonance imaging (MRI) and an assessment conducted by a speech-language therapist that included the narrative task. The participants' vision was checked during the clinical/neurological evaluation to ensure normal or normal corrected vision. Diagnoses were made in meetings coordinated by a senior board-certified psychiatrist (PM) and were based on clinical, neuropsychological and MRI assessments collected by a multidisciplinary team of neurologists, neuropsychologists and speech-language therapists. The participants were then divided into three diagnostic groups: Control, a-MCI, and AD. For the a-MCI variable, the Winblad et al. (2004) criteria were adopted. Memory impairment was objectively defined as performance below 1.5 SD for age on delayed recall tasks of the WMS-III Logical Memory and Visual Reproduction subtests. For the AD variable, we used the diagnostic criteria from the fourth revised edition of the Diagnostic and Statistical Manual of Mental Disorders (DSM-IV-TR) (American Psychiatric Association, 2000) and the National Institute of Neurological and Communicative Disorders and Stroke and the AD and Related Disorders Association (NINCDS-ADRDA) (McKhann et al., 1984).

#### Procedures

The complete language evaluation included a narrative discourse elicited with visual stimuli, confrontation naming (Boston Naming Test – Short Form) and verbal fluency (semantic and orthographic) tasks (Bertolucci et al., 2001). For the narrative discourse task, the participants were shown the same picture cards as the pilot group and were given the same instructions. The same researcher (a speech-language pathologist) assessed all of the participants and was blinded to their pathological status. The task was always administered in the same sequence. All of the sessions were videotaped, and the participants' verbal output was transcribed for further analysis. All of the narratives were then analyzed in accordance with the fixed set of parameters. These parameters were based on previous findings (Davis and Coelho, 2004; Duong et al., 2005; Ska and Duong, 2005; Coelho et al., 2012) and the results of the pilot study. We proposed the effectiveness of speech index as a new parameter. For better control of the variables, we presented the tests in the same order to each participant. The parameters are described below.

- *Narrative time:* The total time in seconds required to perform the narrative task, excluding the latency period between the examiner's verbal directions and the beginning of the participant's narrative.
- *Total number of words:* The automatic word count function of Word software was used, and repeated words were included in the counts. Open-class and closed-class words (these were of limited number and included articles, conjunctions, prepositions, and quantifiers) were counted separately. For open-class words, words that were repeated once or more per sentence were not counted. A separate index was created for this purpose.
- *Discourse type:* Two parameters were established: (1) predominantly narrative and (2) predominantly descriptive. The discourse was considered descriptive when there were no elements of cohesion between the sentences and the narrative comprised frame-by-frame descriptions and the use of present tense or adverbial expressions, such as “here,” or demonstrative pronouns, such as “in this frame.” The discourse was considered narrative when the story was treated as an event that had already occurred, with temporal ordering and causal links between facts. Because some individuals presented a “mixed” discourse with both descriptive and narrative characteristics, we defined narrative discourses as those in which at least 50% of the text presented narrative characteristics. This quantitative criterion was defined by the authors because the literature lacked relevant guidelines.
- *Overall coherence:* We analyzed the number of semantic propositions (macropropositions and micropropositions) following the proposal of Ska and Duong (2005) but did not examine the grammatical complexity of the discourse. Based on Kintsch and van Dijk (1978), this study considers macropropositions as the central idea of each context of action; they are the key feature that determines whether the participant understood the narrative's global meaning and could represent it with a coherent narrative production. Our analysis was based on the nine macropropositions defined in our pilot study. The total number of macropropositions per participant was counted; the maximum was nine main ideas. The number of micropropositions, relevant or irrelevant to the context, was also counted. Micropropositions were defined as information given in addition to the central ideas of the scene (details). All of the ideas that were part of the scene's context were considered relevant, regardless of whether they were essential. Only incorrect or out-of-context ideas were considered irrelevant micropropositions.
  Example:
    *Macroproposition: “The boy set the car's parking brake” (main idea)*.
    *Relevant microproposition: “His sister, who was wearing a blue shirt, leaned her head on the seat and waved to her mother” (actual data – details)*.
    *Irrelevant microproposition: “When it rains, it is not possible to drive” (the statement has no meaning in the context of the action or to the story as a whole)*.
- *Referential cohesion:* Following principles A, B, and C of Chomsky's binding theory (Leitão, 2005), referential cohesion was defined as the use of cohesive elements between sentences during the narrative. It is indicated by the presence of transitional elements and the use of anaphoric co-reference. The “repeated-name penalty (RNP)” phenomenon (Leitão et al., 2012), which occurs when the narrator uses autonomous referents instead of co-reference, was also considered. In this case, RNP penalizes the listener and/or requires him/her to share the speaker's knowledge because the length of the speaker's sentences makes comprehension effortful. Two parameters that encompass the previously mentioned linguistic criteria and the adapted criteria of Davis and Coelho (2004) were defined: (1) appropriate, when the reference elements were used correctly, and (2) inappropriate, when transitional elements were omitted, when there were errors or ambiguities in relation to Principals A, B, or C or when RNP occurred. Only responses classified as narrative were selected for analysis because the cohesion aspects are less observable in descriptive discourse.
  Example of inappropriate referential cohesion:
    “The man is driving. At a certain point, she got out of the car. The passenger who was in the backseat threw himself forward; then the boy who was in the backseat released the parking brake.”
    *(The use of the pronoun* ***she*** *does not make appropriate reference to the antecedent* ***man*.** *In the next sequence*, *the nouns* ***passenger*** *and* ***boy*** *are used as autonomous referents to signify the same subject, which denotes an RNP)*.
- *Index of discourse effectiveness:* This index was obtained by dividing the total number of words recalled by the number of macropropositions. The use of fewer words with more macropropositions indicates a more effective discourse. We proposed this index because it is already known that patients with AD could use many words without using a coherent discourse (Ash et al., 2007). We intend to observe if there is a relationship between the numbers of macropropositions and the total number of words evoked for each group.
- *Narrative structure:* The ability to elaborate the content of the story from a sequence of events that form a full episode was evaluated. The criteria of Stein and Glenn (1979) adopted by Coelho et al. (2012) for evaluating discourse production in patients with TBI were used. The story was considered complete when it involved three aspects: (a) *initial event*, (b) *story development*, and (c) *outcome*. The story was considered incomplete when it involved only one or two of these aspects:
  1. *Initial event: The explanation of the characters and initial action (characterized by the presence of the first and/or second macropropositions)*.
  2. *Story development: The unfolding of the episode, the intentions of the characters and the flow of facts (characterized by the presence of the third to the eighth macropropositions)*.
  3. *Outcome: The direct consequence of the actions of the characters and the conclusion of the episode (characterized by the presence of the ninth macroproposition)*.

#### Statistical analysis

None of the variables of interest exhibited normal distribution (according to Kolmogorov–Smirnov and Shapiro–Wilk tests), with the exception of phonemic and semantic verbal fluency. Therefore, the non-parametric Kruskal–Wallis test was used for continuous variables (i.e., Items A, B, D, and F of the narrative assessment and naming test). *Post-hoc* analysis was performed for all pairwise comparisons with Mann–Whitney (*U*) test (with Dunn–Bonferroni corrections). ANOVA with the *post-hoc* Bonferroni test was used for the parametric variables. The statistical significance was set at 0.05. As required, we performed a complementary analysis of our test results, with level of education as a covariant. We carried out non-parametric ANCOVA – Quade's rank analysis of covariance (Quade, 1967) for non-parametric variables (**Tables 2**, **3**) and parametric ANCOVA for parametric variables (**Table 3**). For categorical variables (i.e., Items C, E, and G), the chi-square and Fisher's exact tests were used. Because the chi-square test does not indicate which cell is responsible for the significant difference, the adjusted standardized residuals were used. Residuals >1.96 indicated statistically significant association among the categories. For all statistical tests, we adopted a level of significance (*α*) of 0.05 (two-tailed). The Statistical Package for the Social Sciences (SPSS) software, version 20.0, was used for all of the analyses.

## Results

There was no significant difference among the groups regarding gender and age. As expected, the global cognitive level of the AD group was significantly lower than that of the Control and a-MCI groups. We used the Geriatric Depression Scale (Brief-form) to perform the screening for depression (exclusion criteria – see Section Sample). The means of the three groups included in this sample did not differ (*p* = 0.600). In addition, the means of all of the groups were below the suggested cut-off for significant depression symptomatology: Control group (2.9 ± 3.09), MCI group (3.52 ± 2.29), and AD group (3.69 ± 3.37). The educational levels of the Control and a-MCI groups were significantly higher than those of the AD group (Table 1). Therefore, we performed an additional analysis with education (years of schooling completed) as a covariant. The significance found previously was maintained for almost all variables (Tables 2, 3). It was possible to observe the effect of schooling in two linguistic variables: phonemic verbal fluency and total number of words – close class, which will be discussed later.

**Table 1:** **Demographic and global cognitive comparisons of the Control and patient groups**.

	**Control Mean (SD)**	**a-MCI Mean (SD)**	**AD Mean (SD)**	**Comparisons**
*N* (total)	41	22	14	
Gender (F/M)	26/15	11/11	10/4	Control ≈ MCI ≈ AD
Age	69.6 (5.8)	72.1 (4.4)	73.4 (7.3)	Control ≈ MCI ≈ AD
Years of education^*^	14.5 (2.6)	13.1 (2.3)	12 (3.3)	Control ≈ MCI > AD
MMSE (0–30)^*^	27.2 (2)	26.2 (1.9)	22.7 (3.5)	Control > MCI > AD

* *Globally significant differences (Kruskal–Wallis)*.

**Table 2:** **Comparison of narrative discourse among the groups**.

***N* (total)**	**Control Mean (SD) 41**	**MCI Mean (SD) 22**	**AD Mean (SD) 14**	Kruskal-Wallis *p*-value < 0.05^*^	**Comparisons**	ANCOVA *p*-value < 0.05^**^
Total words	97.7 (43.2)	117.4 (46.9)	128.0 (61.2)	0.072		0.234
Words – open class	50.2 (21.8)	60.9 (30.4)	57.93 (25.5)	0.225		0.413
Words – closed class	45.2 (21.6)	54.9 (23.3)	64.5 (40.0)	^*^	Control > AD	0.115
Repeated words	2.1 (1.7)	4.2 (3.6)	6.1 (5.8)	^*^	Control > MCI	^**^
Narrative time (s)	65.6 (30.1)	71.4 (34.2)	86.3 (39.2)	0.147		0.280
Total macropropositions	7.6 (1.1)	6.8 (2.0)	4.4 (2.0)	^*^	MCI > AD	^**^
Total micropropositions	1.6 (1.5)	3.1 (3.0)	5.0 (3.7)	^*^	Control > MCI	^**^
Irrelevant micropropositions	0.27 (0.7)	1.1 (1.9)	2.5 (3.8)	^*^	Control > MCI	^**^
Index of discourse effectiveness	13.0 (7.2)	19.6 (12.4)	47.4 (56.9)	^*^	Control > MCI > AD	^**^
	**Control 41**	**MCI 22**	**AD 14**	Chi-square *p*-value^*^	**Fisher's test contrasts**	
Type of discourse: Narrative/descriptive	38/3	19/3	7/7	^*^	MCI > AD	
Narrative structure: Complete/incomplete	34/7	12/10	4/10	^*^	Control > MCI	
Referential cohesion: Adequate/inadequate	(*n* = 38) 30/8	(*n* = 19) 12/7	(*n* = 7) 2/5	^*^	MCI > AD	

* *Significant differences*.

** *Quade's rank analysis of covariance with years of education (non-parametric ANCOVA)*.

**Table 3:** **Comparison of language variables among the groups**.

***N* (total)**	**Control Mean (SD) 41**	**MCI Mean (SD) 22**	**AD Mean (SD) 14**	*p*-value < 0.05^*^	**Comparisons**	**ANCOVA *p* < 0.05**
Naming	14.2 (1.0)	13.4 (1.3)	11.7 (2.3)	^*^	Control > MCI > AD	^***^
Phonemic verbal fluency, F.A.S (total sum)	40.8 (18.7)	33.7 (16.4)	24 (10.7)	^**^	Control > AD	0.103
Semantic verbal fluency, animals (total)	18.1 (4.5)	15.2 (4.6)	9.6 (5.3)	^**^	MCI > AD	^****^

Significant differences

* *Kruskal–Wallis*.

** *ANOVA*,

*** *Non-parametric ANCOVA – Quade's rank analysis of covariance with years of education*,

**** *parametric ANCOVA – Covariance with years of education*.

### Narrative evaluation results

Table 2 summarizes the comparison between the groups regarding narrative discourse. The Control and AD groups differed significantly in nearly all narrative measures, and the a-MCI group's performance represented an intermediate stage between the Control and AD groups for most of the variables.

The index of discourse effectiveness was the only parameter that differentiated the Control, a-MCI, and AD groups. Comparatively, the AD group displayed the worst performance, using more words to generate fewer macropropositions.

For the lexical aspect, there was no significant difference among the three groups regarding the total number of recalled words. A detailed analysis of the type of recalled and repeated words revealed that the three groups did not differ in their evocation of open-class words (*p* = 0.22). The Control group recalled a lower number of closed-class words than the AD group. However, the a-MCI group did not differ from the other two groups. According the posterior ANCOVA analysis, we could see the effect of education level in this specific variable. The covariance showed that the three groups did not differed in total number of closed-class word recalled (Table 2). The Control group used more repeated words in the same sentence than the a-MCI and AD groups did. There were no group differences regarding the time taken to tell the story.

Regarding the macrolinguistic aspect, the AD group's performance differed significantly from that of the Control and MCI groups in terms of global coherence: a smaller number of macropropositions were composed. The AD and MCI groups exhibited similar performances, using more micropropositions and irrelevant micropropositions than the Control group did (*p* < 0.05).

The discourse type was predominantly narrative for the Control (92.65%) and a-MCI (86.3%) groups, which did not differ significantly (*p* = 0.413). Both groups differed from the AD group (*p* < 0.01), in which 50% of the individuals produced predominantly descriptive discourses.

The Control group performed better on the development of the narrative structure, completing 83% of the narrative episodes. The Control group differed (*p* < 0.03) from the a-MCI and AD groups, which provided 54.5 and 28.5% completed narratives, respectively; the a-MCI and AD groups did not differ from one another (*p* = 0.176).

The Control and a-MCI groups did not differ in their use of referential cohesion elements (*p* = 0.220). A total of 79% of the individuals in the Control group and 63.1% of the individuals in the a-MCI group did not commit any errors in explicit referents and anaphoric co-reference use. None of the individuals in the Control group displayed the RNP phenomenon. The AD group performed worse than the Control and a-MCI groups (*p* < 0.02); only 28% of their narratives showed adequate cohesion. The most common errors in the AD group were omissions of the explicit referent, inadequate or ambiguous use of pronouns to characterize the antecedent and the RNP phenomenon.

Table 3 summarizes the comparison among the groups regarding their usual language variables. The Control and AD groups differed significantly in all measures, as expected. The a-MCI group only differed from the Control group on a naming test. In both semantic and phonemic verbal fluency, these two groups had a similar performance. a-MCI and AD had a significant difference in semantic verbal fluency but not in phonemic verbal fluency. ANCOVA showed the effect of education level to differentiate Control and AD groups in phonemic verbal fluency test; previous significance (ANOVA) between these two groups is no longer seen.

## Discussion

The objective of the present study was to introduce parameters for evaluating narrative discourse based on a sequence of action pictures that were visually presented to elderly people with and without cognitive decline. Because of the ceiling effect that may result from the use of simpler tasks, a more complex task, such as the one used here, might be more suitable for determining the difficulties that patients experience during the early stages of cognitive decline (Forbes-McKay and Venneri, 2005). In addition, narrative discourse in everyday's life requires the speaker to verbally reproduce an episode experienced in the past and the investigation of deficits in MCI and AD may provide more insight into the autobiographical memory deficits already described in those disorders (Urbanowitsch et al., 2013).

The proposed task offers some advantages for clinical use in language assessment batteries. The major advantage is that it decreases the influence of episodic memory on linguistic performance. Other advantages include the analysis of micro- and macro-linguistic aspects using a single ecological activity that takes advantage of individuals' familiarity with the context of the action and the relatively easy and quick administration procedures (approximately 5 min).

The second and main objective of this study was to assess the applicability of this task for differentiating among healthy controls, people with a-MCI and people with AD.

### Performance differences between the control and AD groups

The results indicated that the Control group differed from the AD group in nearly all of the analyzed macro- and micro-linguistic parameters. However, the total number of words recalled and the time required to produce the narrative could not discriminate any of the three groups. Although many individuals from the Control group also used an excessive number of words, extended the narrative and provided comments about the scene, they produced an effective discourse with the expected macropropositions and a complete narrative structure. In contrast, regardless of the number of words used, the individuals in the AD group had great difficulty presenting a structured and semantically appropriate narrative discourse. These results suggest that the lexical-semantic deficit alone does not adequately explain the macrolinguistic discursive problems in AD individuals.

The AD group displayed difficulties with global coherence, produced fewer macropropositions and less complete stories and exhibited lower discourse effectiveness compared with the Control and a-MCI groups. Thus, analyzing the number of words and the time required for the narrative is less important than analyzing the relationship between the words and the number of macropropositions generated and the ability to structure the full narrative episode.

The significant differences between the Control and AD groups are corroborated by other studies that reveal a decline in narrative discourse in AD (Ska and Duong, 2005; Ash et al., 2007; Taler and Phillips, 2008; de Lira et al., 2011). In our study, the AD group consisted of individuals with CDR scores of 0.5 or 1.0, which emphasizes that this linguistic impairment is already present during the disease's initial stages (Chapman et al., 2002). The phonological and basic syntactic structuring aspects were not the focus of our study because they are not expected to decline in early AD.

Some authors argue that discursive difficulties might be related to episodic memory and executive deficits (Carlomagno et al., 2005; Cannizzaro et al., 2012). Given that the test used in this study does not overload episodic memory, our findings might reinforce the hypothesis that the coherence and cohesion difficulties observed in AD individuals are associated with the executive and semantic-pragmatic components of language. Semantic memory is required for pre-verbal planning and, along with working memory, promotes top-down processing. Working memory is also required during verbal assertion (Caselli et al., 2014).

In a study on a French population that used the same task, Ska and Duong (2005) also observed less complete discourses with decreased content in the AD group compared with a group of healthy elderly. The authors explained that these findings differ for narrative tasks elicited with visual sequences and description tasks, in which individuals can maintain the primary content even if they do not clearly describe details or complementary information. Only narrative discourse demands an integration of successive events and the relationship between such events and previous knowledge to formulate the meaning of the story to be narrated.

Although the visual stimuli remained available throughout the entire narrative time, the participants were instructed to carefully observe the depicted sequence of actions before starting the narrative. To elaborate the pre-verbal content, the individual must rely on sociocultural and discursive models previously stored in long-term memory. Subsequently, the beginning of the structuring process and the use of cohesive elements during the narrative require working memory because completely elaborating the narrative requires maintaining and updating the narrated sequence until the story ends.

In a study comparing AD patients, aphasic patients and a control group, Carlomagno et al. (2005) argued that individuals with AD had greater difficulty integrating information and mentally representing discourse formulation. The author suggested that decreased working memory in association with semantic-pragmatic difficulties could also explain the predominance of descriptive discourse and verbal productions with absent or erroneous referential cohesion. In our sample, the AD group presented similar patterns, such as the omission of transition elements, the absence of explicit referents and an excess of inappropriate or ambiguous personal pronouns in relation to the prior referent. Those issues might be caused by lexical retrieval difficulties during the discourse formulation stage and by working memory impairment. It is possible that the inadequate use of cohesion elements, which affects discourse coherence, is caused by a problem with the interface between the semantic-pragmatic component and working memory. It is important to note that is very difficult to demarcate the boundaries between this cognitive processes and verbal language abilities, such as generating narratives.

### The MCI group's performance

The a-MCI group's results represented an intermediate performance between the Control and AD groups for most of the studied parameters. For some tasks, their performance was similar to that of the Control group; for others, their performance was similar to that of the AD group, as described below.

The linguistic pattern of the a-MCI group resembled that of the Control group in terms of the type of discourse used, the coherence and the cohesion. The production of predominantly narrative discourse, the ability to establish the global coherence of the story with the use of more macropropositions and the production of a more effective discourse than the AD group might indicate that these macrolinguistic aspects of narrative were less impaired in a-MCI patients.

The good performance of the a-MCI group in these aspects might be related to two factors: the low use of episodic memory during the task (cognitive deficits are a characteristic of the a-MCI group) and the proper functioning of working memory (Troiani et al., 2008; Cannizzaro and Coelho, 2013). However, the a-MCI group used more irrelevant micropropositions and propositions than the Control group, and their performance was more similar to that of the AD group. We found no studies of this population and task; therefore, we had no basis with which to compare these results. Given that a-MCI is a transitional stage between senescence and AD, we hypothesized that the first manifestations of the macrolinguistic plan in the narrative discourse in this group could be intrusions of information, particularly information that is irrelevant to the story's context. This hypothesis was based on the fact that individuals in senescence provide additional information in their discourse, though without using inadequate propositions. In addition to the exacerbated presence of relevant and irrelevant micropropositions, the AD group displayed more global decline, with an absence of the macropropositions that were needed to properly construct the narrative.

Considering that there was no interference from episodic memory in the construction of discourse and in top-down planning and that adequate generation of macropropositions was present, we may hypothesize that such additions of unnecessary information and the introduction of out-of-context elements by the a-MCI group might represent problems with the semantic-pragmatic component of language. In addition, the a-MCI group had difficulties completing the story; 45% of the individuals produced incomplete narratives, most often without providing a necessary description of the story's outcome.

In the microlinguistic plan, the a-MCI and AD groups displayed similar performances in relation to the mean number of repeated words in the same sentence. This aspect differentiated these groups from the Control group, in which such repetitions rarely occurred. This finding may illustrate the need to rephrase ideas when structuring the assertion, an act that is an expected part of the discourse among the AD population (Mansur et al., 2005) but that has not been described in a-MCI.

### Differences among the three groups

The discourse effectiveness index is obtained by dividing the total number of words recalled by the number of macropropositions generated. It has been proposed for analyzing the efficiency of the discourse. This discursive measure was suitable to differentiate the three groups, and it involves the relationship between lexical assessment and the semantic-pragmatic domain in the context of narrative. A larger number of words does not necessarily provide more macropropositions. There was no difference between the total number of words and open class words among the three groups; however, there was a large difference in the number of macropropositions generated.

Aspects related to naming and lexical evocation could be analyzed independently of the context of the narrative form. There are controversial studies in the literature regarding the effectiveness of these tests for discriminating control and MCI groups. In our sample, the naming test differentiated the three groups; this differs from the semantic verbal fluency tests, which could only discriminate between the control and AD groups (Table 3), and the phonemic verbal fluency test, which could differentiate the control group from the a-MCI and AD groups. After we performed the covariance analysis with years of education this difference between control group and AD group in phonemic verbal fluency disappears, indicating the effect of education in this task. Such result is consistent with other studies that have shown the influence of sociodemographic characteristics such as age and education level (Senhorini et al., 2006). Others however (Steiner et al., 2008) have demonstrated that age but not the level of education has the largest effect on that task.

It is important to consider that analysis of covariance using the quantitative education (years of schooling completed) should be carefully analyzed. Such analysis has been criticized because it does not allow control over recency or quality of education (Miller, 2013). This aspect is particularly important for the Brazilian population where there is great heterogeneity in the quality of formal educational provided.

### Limitations

This study has some limitations. First, the sample of the pilot study was relatively small. The psychometric properties of the task are presently being investigated. Although the overall objective of this study was to investigate the continuum of language deficits from healthy individuals to those with a-MCI and AD using a task that did not make episodic memory demands, our a-MCI group comprised individuals with single and multiple domain subtypes; we did not control the influence of such heterogeneity because of our small sample size. We analyzed the effect of educational level in different linguistic variables but we did not analyze the effect size of this influence for each variable because it was beyond of the scope of this study, but it should be considered in future research. It is important to ponder that narrative tasks elicited by visual stimuli may require increased visual perception, which must be evaluated during clinical evaluation of the patients. In addition, it is possible that the scenes themselves could be used as supports, which could result in a more descriptive discourse. Such tendencies must be minimized during instruction. Visual integration and other cognitive domains, such as executive function, are interrelated, and tasks designed to evaluate one specific cognitive aspect must consider other potential contributors to performance; future studies might include a regression analysis.

## Conclusions

In conclusion, narrative discourse elicitation with visual stimuli is useful for analyzing language in elderly people with cognitive decline, including a-MCI and AD. The task was suitable for differentiating the Control and AD groups, and it showed that the a-MCI group exhibited discursive deficits compared with the Control group. This task has advantages. In addition to being an ecological assessment, it allows a characterization of the subtle and complex language skills (semantic-pragmatic processing) that are initially affected during the early stages of AD, such as a-MCI. The influence of the educational level in this kind of study must be considered.

## Author contributions

CD and GC contributed equally to this study. CD, GC, PM, and RF designed the study. CD, GC, AT, and NA conducted the experiment. CD and GC analyzed the data. CD, GC, RD, RF, JM, PM, and FT contributed to the manuscript.

### Conflict of interest statement

The authors declare that the research was conducted in the absence of any commercial or financial relationships that could be construed as a potential conflict of interest.
