# Supplementary material for: Deficits in narrative discourse elicited by visual stimuli are already present in patients with mild cognitive impairment
Source: Front Aging Neurosci. 2015 May 28;7:96. doi: 10.3389/fnagi.2015.00096 (PMC4446997; doi:10.3389/fnagi.2015.00096)
Supplement: Supplementary file 1 [file DataSheet1.DOCX]

**Appendix 1. List of the nine macropropositions and keywords by scene.**

1. The **mother** was driving the **car** with two kids (scene 1).
2. She **stopped** the **car, irresponsibly**, to **ask** for **help** or **do something** (scene 2).
3. She **left two children** alone **screaming inside** the **car** (scene 2).
4. **The mischievous boy moved to the front seat** (scene 3).
5. He **released the** **parking brake** or **moved** the **steering wheel** to **drive** (scene 4).
6. Without brakes, **the car** **went down the hill** (scene 5).
7. **It hit** the **light post** (scene 6).
8. The **mother saw** what **happened** and **came running** desperately **to help** the **children** (scene 6).
9. She **gave** the **kids** a good **scolding** and returned **home** with **the crashed car** or **went to fix it** (scene 7)

**Words or phrases present in various transcriptions and accepted as replacements for the keywords:**

Mother: driver, woman, lady

Two children: her children, the kids

Stopped: parked

Do something: buy something, go to a store, make a purchase, look for something

Inside the car: in the back seat

Moved: jumped, leaned

Mischievous: misbehaved, naughty, playful, wicked

Released the parking brake: moved, played with, let the brake go

Went down the hill: began to move, went straight

Desperately: scared, nervously, anxiously, angrily, guiltily

Gave a good scolding: got mad, screamed

Returned home: left, went to the mechanic

Crashed: broken, damaged

**Appendix 2. List of grouped keywords and their factor loadings (by scene) based on CFA.**

**
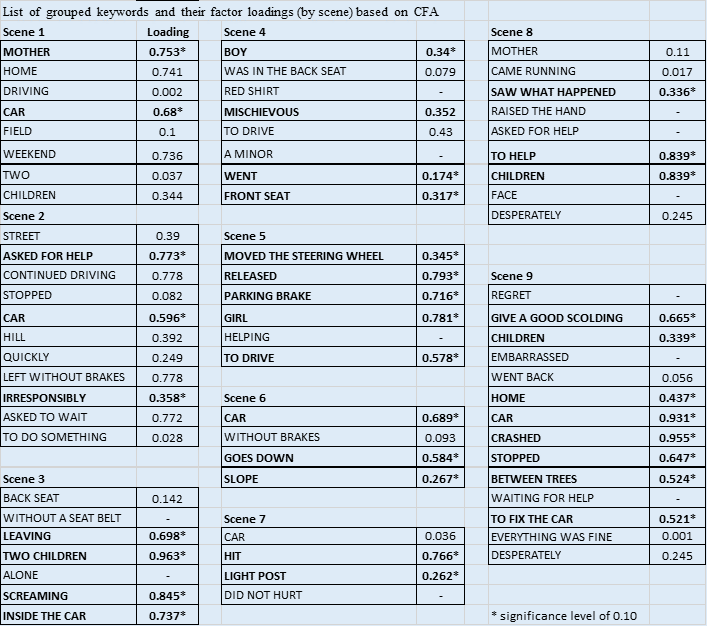
**
